# Impacts of Metals on Infectious Diseases in Wildlife and Zoonotic Spillover

**DOI:** 10.3390/jox15040105

**Published:** 2025-07-03

**Authors:** Joel Henrique Ellwanger, Marina Ziliotto, José Artur Bogo Chies

**Affiliations:** Laboratory of Immunobiology and Immunogenetics, Postgraduate Program in Genetics and Molecular Biology (PPGBM), Department of Genetics, Universidade Federal do Rio Grande do Sul (UFRGS), Porto Alegre 91501-970, Rio Grande do Sul, Brazil; marinaztto@gmail.com (M.Z.); jabchies@terra.com.br (J.A.B.C.)

**Keywords:** infectious diseases, ecoimmunology, disease ecology, immunology, metals, nutritional ecology, parasitology, pollution, toxicology, wildlife

## Abstract

Climate change, mining activities, pollution and other human impacts on the natural environment cause significant changes in the concentrations and mixtures of metallic elements found in different ecosystems. Metals such as cadmium, copper, lead and mercury affect multiple aspects of host–pathogen interactions, influencing the risk of infectious diseases caused by various classes of pathogens. Notably, exposure to metals in doses and combinations toxic to the immune system can favor the dissemination of pathogens in natural environments, threatening the reproduction, well-being and survival of varied animal species. However, these problems remain neglected, since the influences of metals on infectious diseases are studied with a primary focus on human medicine. Therefore, this article aims to review the influence of metals/metalloids (e.g., arsenic, cadmium, chromium, copper, iron, lead, mercury, nickel, zinc) on infectious and parasitic diseases in animals living in natural environments. The potential impact of metals on the risk of zoonotic spillover events is also discussed. Metal pollution tends to increase as the demand for elements used in the manufacture of industrial products, batteries, and electronic devices increases globally. This problem can aggravate the biodiversity crisis and facilitate the emergence of infectious diseases. Considering the interconnections between pollution and immunity, measures to limit metal pollution are necessary to protect human health and biodiversity from the risks posed by pathogens. This review helps fill the gap in the literature regarding the connections between metal pollution and various aspects of infectious diseases.

## 1. Introduction

Metal pollution has anthropogenic and natural (geogenic) causes [1,2]. Metal emissions from natural events, such as volcanic activity, rock weathering, and wildfires in fire-dependent ecosystems, are beyond human control [1,3]. The impossibility of totally controlling natural events and their consequences makes it even more crucial to limit the anthropogenic emissions of metals to reduce their impact on the environment [3]. Metal pollution has become a problem of increasing concern as human impacts on the environment intensified from the 1950s onwards [4], especially considering the intensive mining and agricultural practices (e.g., the use of fertilizers and pesticides containing metals), poor management of electronic waste and batteries, increasing industrial processes, and release of metals from automotive components and traffic (e.g., tire, fuel, road runoff), among other anthropogenic sources and activities [1,5,6,7]. Metal pollution currently affects the soil on every continent, putting between 0.9 and 1.4 billion people at risk of suffering severe health consequences from exposure to metals [2].

The growing demand for electronic devices and efforts towards a green energy transition indicate an upward trend in mining activities globally [8]. Industrial and artisanal mining are responsible for a large portion of the metal pollution observed in several places around the world, either due to by-products or mining procedures or due to accidental spills and other mining-related disasters. In Brazil, the rupture of two mining tailings dams in the state of Minas Gerais in 2015 (Mariana city) and 2019 (Brumadinho city) led to the contamination of different ecosystems with several toxic metals [9,10]. In the Amazon region, artisanal gold mining causes mercury (Hg) contamination of forest ecosystems, leading to the bioaccumulation of this metal in fish [11]. In addition to these metal pollution-related case studies located in Latin America, Table 1 presents other examples [12,13,14,15,16,17,18,19,20,21,22] of pollution associated with mining activities in various regions of the world, showing that this is a problem on a global scale. Artisanal battery recycling in Africa, Latin America and Asia is also responsible for releasing large amounts of metals into the environment [23]. Furthermore, climate change and extreme weather events affect the distribution, concentration and bioavailability of metals in soils, water bodies and the atmosphere [24].

The mixtures and concentration of chemical elements observed in ecosystems, whether caused by human actions or by natural geographic variations in soil elemental concentrations, may have important effects on human infectious diseases. For example, it has already been hypothesized that the levels of selenium (Se), a non-metal element, found in soils in different regions of the world influence the risk of viral infections in human populations [25]. Also, it was demonstrated that the amount of the metal vanadium (V) present in water sources from Hawai’i increases the risk of pulmonary bacterial infections in humans [26].

While the impact of metals on human infectious diseases is better established [27,28], there is limited data on their effect on infection risk in nature, especially concerning non-human vertebrates [29]. Evidence since the 1980s highlights the significant influence of environmental metals on animal infection rates, as discussed by Kosoy and Biggins [30]. Also, it is well known that the levels of metals present in soils may affect wildlife health status and the risk of infectious diseases in animals, especially those that live in direct contact with the soil, such as earthworms, snails and insects [31]. However, this study topic remains overlooked in the field of disease ecology. Thus, the aim of this article is to review the influences of metals on infectious diseases in animals living in natural environments, including birds, mollusks, non-human mammals, reptiles, fish and bees. Also, how these influences affect the risk of zoonotic spillover events will be discussed.

## 2. Methodological Notes

This is a narrative review intended to highlight a gap in the literature regarding the effects of metal exposure on infection-related outcomes in non-human animals. The documents included in this review come from non-systematic searches in PubMed [32] and Google Scholar [33] in 2024 and 2025 using keywords such as “metal”, “infection”, “infectious diseases”, “wildlife”, “environment”, “animal”, “spillover” and “zoonosis” in different combinations. The literature cited in the selected documents was also used as a research source. Finally, the authors’ virtual library was consulted for supplementary sources relevant to the topics addressed in this review. Considering the scarcity of references on the effects of metals and infectious diseases in natural environments, no limitation was applied regarding the year of publication of the works for inclusion in the review. In addition to studies carried out with animals sampled in nature, some experimental and theoretical studies were included in the review as they provide relevant information to explain the mechanisms of metal toxicity and their relationship to infectious processes.

Section 3 shows a summarized view of the effects of metals on animal infection. Sequentially, the detailed results of studies evaluating the impacts of metals on infections in invertebrates such as bees (Section 3.1) and mollusks (Section 3.2), and vertebrates such as fish (Section 3.3), reptiles (Section 3.4), birds (Section 3.5) and non-human mammals (Section 3.6), is presented and discussed. A synthesis of the mechanisms involved in the influence of metals on infectious diseases is presented in Section 4, and the potential impacts on zoonotic spillover events are described in Section 5. A general discussion is presented in Section 6. Finally, Section 7 presents a summary of the main results of the reviewed articles, concluding the article.

## 3. Impacts of Metal Exposure on Infection-Related Outcomes in Animals

Table 2 summarizes the main effects of metals on infections in animals sampled in nature based on the main studies [29,34,35,36,37,38,39,40,41,42,43,44,45,46,47,48,49,50,51,52] reviewed in this article. The following sections provide more detailed descriptions of these studies, supplemented by a discussion covering additional field, theoretical and laboratory investigations.

### 3.1. Impacts on Insects (Bees)

Invertebrates may be exposed to metal pollution through contact with the environment (e.g., earthworms through metals present in soil) or through food chains (e.g., phytophagous insects through metals present in plants) [53,54]. Metals affect microbe–insect interactions by regulating insect immune systems, affecting the host microbiome, promoting physiological stress and regulating the expression of genes that act in defenses against pathogens, among other mechanisms [54,55,56,57].

Szentgyörgyi et al. [34] investigated the accumulation of metals (i.e., lead (Pb), cadmium (Cd), zinc (Zn)), species diversity and parasite load (Microsporidia *Nosema bombi*) in bumblebees. The study was performed in Poland and Russia, considering different sites and including three metal-polluted locations (i.e., Guryevsk metallurgic plant (Russia), Belovo Zn smelter (Russia) and Olkusz Zn smelter (Poland)) and two control regions (the mountains of Kouznetskiy Alatau and Gornaya Shoria, both in Russia). The levels of Pb and Cd in bumblebee tissues correlated with the content of these metals detected in soil samples from the Guryevsk and Belovo locations. A significantly higher percentage of infected bees was found at the Kouznetskiy Alatau control region (29.4%) compared to a specific site in Guryevsk (6.1%). However, the metal pollution indexes did not significantly impact species diversity parameters or correlate with infection rates, as reported by Szentgyörgyi et al. [34]. Further studies evaluating infection rates by other microorganisms in bees and other insects exposed to different levels of metal pollution are needed to determine the intensity with which this animal group is affected by metals and how this is reflected in infection outcomes.

### 3.2. Impacts on Mollusks

Analyzing cockles (*Cerastoderma edule*) collected at Banc d’Arguin, south-west France, Baudrimont et al. [36] found no significant difference in levels of Cd, Pb, mercury (Hg), Zn and copper (Cu) between bivalves parasitized with *Labratrema minimus*, a digenean parasite, and those without infection. Notably, metal concentrations were generally very low, likely due to the mollusk collection site being relatively unpolluted, as suggested by the low content (<2%) of organic matter [36]. In Ireland, a study evaluated *Littorina littorea* periwinkles that were also infected with digenean parasites and found reduced levels of iron (Fe), Cu and nickel (Ni) compared to uninfected periwinkles. Obliteration of the hepatopancreas caused by digenean parasites may result in decreased metal storage, potentially explaining these results [35]. Also, digenean parasite infection can modulate metallothionein synthesis in mollusks, impacting metal metabolism by altering the detoxification of toxic elements and the homeostasis of essential metals [36].

Some parasites accumulate large amounts of metals [58]; therefore, parasite–metal–host interactions have complex effects on infection outcomes. In a study performed in Egypt, Cd water levels were positively associated with *Schistosoma mansoni* infection rate in the offspring of *Biomphalaria alexandrina* snails, while Pb, Cu and Hg water levels were negatively correlated with infection rate [37]. In an experimental study, *B. glabrata* snails infected with *Schistosoma mansoni* parasites and concomitantly exposed to metals (Cd, Hg and Pb) showed increased mortality rates compared to uninfected snails exposed to metals [59]. Similarly, another experimental study [60] described that co-exposure to Cd and *S. mansoni* affect the growth and reproduction of *B. alexandrina* snails, in addition to reducing snail survival rate. Taken together, these results indicate that metals affect mollusks’ parasitic infection response and can enhance the deleterious effects of pathogens.

### 3.3. Impacts on Fish

In the 1960s, Pippi and Hare [38] reported an epizootic outbreak affecting Atlantic salmon (*Salmo salar*) and suckers (*Catostomus commersonii*) caused by the bacteria *Aeromonas liquefaciens* in Miramichi River, New Brunswick, Canada. The epizootic outbreak was linked to the combined effects of two environmental stressors: river pollution by Cu and Zn and elevated river temperatures. These two factors made the fish more susceptible to infection (i.e., “weakened” immune defenses) and provide conditions conducive to the development of bacteria (i.e., near-optimum temperature), triggering the epizootic outbreak [38]. Indeed, metals can modulate the immune system and physiology of fish, thus affecting disease susceptibility [61].

In a study performed with European chubs (*Squalius cephalus*) from Sava River, Croatia, fish infected with the acanthocephalan *Pomphorhynchus laevis* showed lower levels of Cu, Cd and Pb compared to uninfected fish [40]. In South Africa, infected fish from Hex River showed (non-significant: *p* > 0.05) lower metal concentrations (e.g., Cd, chromium (Cr), Ni, platinum (Pt) and Pb) compared to uninfected fish. It was also observed that fish with a higher infection load had lower levels of metals in their tissues in comparison to fish with reduced infection intensity [41]. Similar results were observed in Egypt, where Hassanine and Al-Hasawi [39] detected reduced levels of Cd or Pb in liver samples of fish (*Siganus rivulatus*) infected with the intestinal acanthocephalan *Sclerocollum rubrimaris* compared to uninfected fish.

Sures and Siddal [62] showed experimentally that acanthocephalan parasite *P. laevis* parasitizing the intestine of chub (*Leuciscus cephalus*) accumulated high Pb content (much higher than those levels detected in host tissues). Marijić et al. [40] detected higher levels of many metals (i.e., Cu, manganese (Mn), silver (Ag), Cd, Pb) in acanthocephalans than those observed in fish gastrointestinal tissue samples. When analyzing parasitized fish, the cestode *Atractolytocestus huronensis* and the nematode *Contracaecum* sp. accumulated higher metal levels than those detected in host tissues [41]. Adding to this evidence are the recent findings of Mijošek et al. [42], which observed higher content of several metals, including the highly toxic Pb, Cd and thallium (Tl), in acanthocephalans (*Dentitruncus truttae*) as compared to those detected in the intestine of fish (*Salmo trutta*) samples at Krka River, Croatia. Inverse relationships between parasite abundance and metal levels in fish were observed for several metals and other elements, specifically As, Cr, Mn, Se, V, molybdenum (Mo), barium (Ba), Cu and Fe. This result reinforces the protective role of parasites against metal intoxication in fish [42].

Parasites can capture bile-bound metal complexes that form in the liver and are released through the bile duct into the intestine, thereby reducing the availability of metals for reabsorption by the host’s intestinal cells. Furthermore, parasites tend to accumulate significantly higher levels of metals compared to those present in their environment. This high bioaccumulation capacity makes parasites good bioindicators of metal pollution. Also, the bioaccumulation capacity of some parasite groups (i.e., acanthocephalans and cestodes) may reduce metal accumulation in fish through metal sequestration. In practical terms, this body of information suggests that, in addition to potentially protecting fish from metal toxicity, parasites may also play a valuable role in environmental monitoring. The abundance and diversity of parasite species in fish, along with the concentrations of metals accumulated in these parasites, can serve as complementary indicators for monitoring metal pollution in biological systems [39,40,41,58,62].

Finally, methylmercury (MeHg+), a toxic form of organic Hg, enters the wildlife food chain primarily through bioaccumulation in fish and shellfish. Therefore, it is not only harmful to fish themselves but also poses a serious threat to birds, mammals and other animal groups that feed on fish and shellfish [3]. Although the entry of Hg into the food chain through fish/shellfish is a well-known problem, with widely reported toxic effects on humans [63], the effects of Hg on infectious diseases are still unknown. Considering the immunotoxic and immunodisruptive activities of Hg [64,65], the potential effects of this element on infectious processes represent an interesting research question.

### 3.4. Impacts of Reptiles

A study performed with *Chalcides ocellatus* lizards collected from industrial, rural and urban sites from Egypt evaluated the prevalence of parasitic infection and the levels of metals (i.e., Cu, Ni, Cd, Pb) in parasite and intestinal lizard tissue samples [43]. One cestode (*Oochoristica tuberculata*) and five nematode species (*Thelandros alatus*, *Parapharyngodon micipsae*, *Pharyngodon mamillatus*, *Spauligodon auziensis* and *Cosmocerca ornata*) were found infecting the lizards. Nematode and cestode infection intensity correlated positively with Cu, Cd and Pb levels at all sites studied. The cestode *O. tuberculata* accumulates more metal levels, particularly Cu and Cd, compared to nematodes. Also, lizards infected with *O. tuberculata* showed increased metal intestinal levels. These findings indicate that *O. tuberculata* may serve as a biomarker for metal pollution [43]. Considering the direct interaction that reptiles have with the environment, especially soil, more research on the effect of metal pollution on the immune system and infectious processes in reptiles is welcome.

### 3.5. Impacts on Birds

Mashima et al. [45] investigated an avian cholera outbreak caused by *Pasteurella multocida* in oldsquaws (*Clangula hyemalis*) in the Chesapeake Bay (USA) in 1994. Their findings indicated that elevated Cd levels in liver and kidney samples were linked to a higher risk of infection. The authors also observed that Se and Hg levels were lower in liver samples from birds that died of cholera compared to control birds [45].

Studying house sparrow (*Passer domesticus*) populations in France, Bichet et al. [29] evaluated whether the concentration of Pb, Cd and Zn in feathers was associated with the infection prevalence of *Plasmodium relictum*, the agent of avian malaria. Infection prevalence was negatively associated with Cd concentrations. Sub-chronic exposure to some metals may have a stimulatory effect on the immune system, potentially explaining this result. Alternatively, this result could be explained by a lethal toxic effect of Cd on the parasite. On the other hand, Pb concentrations were positively correlated with *P. relictum* prevalence, potentially due to the immunotoxic effect of lead on the immune systems of the sparrows. Considering that Pb concentrations were higher in urban/polluted habitats, another potential explanation for the observed results is the increased exposure of these animals to malaria-infected mosquitoes (a valid explanation for areas with urban circulation of malaria parasites) [29]. Also, in France, high levels of Pb in feathers were associated with an increased intensity of Haemosporidian blood infection in *Columba livia*, the urban pigeon [46].

In agreement with these previous studies, a recent work by Khan et al. [44] on house sparrows (*Passer domesticus*) from two islands of Norway (Hestmannøy and Gjerøy) found that increased blood Pb levels were linked to higher infection rates of *Syngamus trachea*, a parasitic nematode commonly known as “gapeworm”, the causative agent of syngamosis, a respiratory tract disease. This result was also attributed to the immunotoxic effect of Pb [44]. The relationship between Pb exposure and increased infection risk observed in nature is reinforced by some experimental data. In a study with red-legged partridges (*Alectoris rufa*), increased experimental Pb exposure showed a season-dependent impairment on T-independent humoral response, decreased levels of natural antibodies and lysozymes, and was non-significantly associated with a higher prevalence of the parasite *Heterakis gallinarum* [66]. Finally, Khan et al. [44] also found that infected sparrows from Gjerøy had higher magnesium (Mg) concentrations and lower levels of V compared to non-infected birds.

In addition to the toxic and immunosuppressive effects of metals, it is important to consider the beneficial effects of some metals, such as Zn, on the immune system and protection from infectious disease. Analyzing urban pigeons (*Columba livia*) from France, Gasparini et al. [46] found that increased Zn levels in feathers were associated with a lower intensity of Haemosporidian parasites in blood as well as a lower infection prevalence of *Chlamydiaceae bacteria*, the causative agent of ornithosis disease. Conversely, higher Pb levels were associated with increased Haemosporidian infection, as mentioned previously [46].

### 3.6. Impacts on Non-Human Mammals

A review of a set of ecological investigations performed in the 20th century by the Russian researcher Evgeny Rothschild showed that the types and quantities of metals observed in nature influence the rate of *Yersinia pestis* infection in free-ranging rodents [30]. This information is corroborated by additional evidence. In the south of Antwerp, Belgium, Tersago et al. [48] observed that wood mice (*Apodemus sylvaticus*) resistance to the endoparasite *Heligmosomoides polygyrus* decreased with increasing exposure to Ag, arsenic (As), Cd, cobalt (Co) and Pb. Notably, the number of *H. polygyrus* was positively correlated with Cd levels measured in host liver samples, and the number of fleas observed in the animals was positively correlated with Pb levels. These results suggest an immunotoxic effect of metals on the immune defenses of rodents [48].

Other mechanisms behind the relationship between metals and infection in rodents have begun to be elucidated more recently. Experimental Cd exposure facilitated *Salmonella* infection in C57BL/6 mice. These results were linked to higher inflammation and damage in the intestinal mucosal barrier, along with other dysfunctions caused by Cd [67]. Also, in an experimental study performed with male Wistar rats, co-exposure to Cd and infection with the intestinal parasite *Moniliformis moniliformis* (Acanthocephala) disrupted hormone homeostasis, potentially harming animals’ health [68].

In a study performed in the Czech Republic, Jankovská et al. [51] found decreased levels of Pb in kidney samples of foxes (*Vulpes vulpes*) infected with the parasites *Toxascaris leonina* (Nematoda) and *Mesocestoides* spp. (Cestoda) compared to non-infected foxes. Similarly, also in the Czech Republic, Brožová et al. [50] reported lower levels of Cd and Pb in the small intestinal tissue of red foxes (*Vulpes vulpes*) infected with the tapeworm *Echinococcus multilocularis* compared to non-infected foxes. In contrast, levels of other metals increased, specifically Cr, Cu, Fe, Mn, Ni and Zn; however, despite marked differences in metal levels between groups, only Fe showed a statistically significant difference [50]. Jankovská et al. [51] also reported statistically significantly increased levels of Mn and Cu in liver samples of parasitized foxes compared to non-infected animals, as well as higher Pb, Mn, Cu, Ni and Zn accumulation in parasites than in host tissues. These results support the capacity of parasites to sequester some metals [51], thereby decreasing their concentrations in host tissues, while remobilizing other elements, increasing their concentrations in the host [50]. Importantly, the taxonomic groups of parasites with which the host is infected (e.g., cestodes or nematodes) may influence the levels of metals in the host tissues, as shown by a study permed with small rodents [49]. In brief, certain parasite species, in natural environments, may confer protection to their hosts against the toxic effects of metals. This finding contributes to a broader understanding of the potential ecological roles of parasites and challenges the common predominantly negative perception of parasites.

In a study performed with carcasses of harbor porpoises (*Phocoena phocoena*) from the coastal waters of England and Wales, Bennett et al. [47] observed increased liver concentrations of Hg, Se and Zn in the porpoises that died of infectious disease compared to porpoises that died from physical trauma (e.g., entrapment in fishing nets), as determined by field data and pathological investigations. Infected animals were considered to be those with evidence of viral, parasitic, bacterial or fungal infections, with parasitic pneumonia associated with secondary bacterial infections being the most frequent observation. The Hg:Se molar ratio was also increased in infected animals compared to controls. The immunosuppressive effects of Hg may explain the increased infection rate, while higher Zn levels may be explained by the redistribution of this element due to infection. However, factors such as age and nutritional status have been identified as potential confounding factors that may complicate the associations between infection status and metal levels. No significant difference was found between the groups concerning liver concentrations of Pb, Cd, Cu and Cr [47]. Finally, we underscore that the impact of metals on infection in non-human mammals may vary across different taxonomic groups. For example, in bats, the effects of Hg on infection risk are varied and complex, differing among species [52].

## 4. Synthesizing the Mechanisms

The immunomodulatory effect of metals is a primary mechanism by which these elements influence infectious diseases, with metal exposure being increased or decreased by geogenic events and anthropogenic activities in natural environments. Human activities that enrich soils with toxic metals, such as mining, can promote immunotoxicity due to the toxic effects of a specific metal (e.g., Hg, As, Pb) or the synergistic effects of multiple toxic metals [24]. On the other hand, soil depletion caused by human activities (e.g., monoculture, deforestation, irrigation, erosion) can contribute to the production of foods deficient in essential metals like Zn and Fe, which are crucial for proper immune function [24]. For instance, Zn deficiency is associated with elevated levels of inflammation and dysregulated immune function, while excess Zn is associated with the suppression of T and B cells and stimulation of regulatory T cells [69].

In brief, the poor functioning of the immune defenses, both due to a deficiency and excess of metals, may contribute to increased host susceptibility to infections [24]. Furthermore, metals can activate or suppress the expression of genes that regulate immune function, affecting both susceptibility to infections and the pathogenesis of infectious diseases [24,69,70]. Recently, using a toxicogenomic approach, we reported that As, Hg and Pb affect the activity of thousands of genes across different species. Many of these genes are involved in infectious processes related to viruses, bacteria and parasites [71].

In addition to immunomodulation, other mechanisms may explain how metals affect the risk of infectious diseases. For example, many microorganisms, such as bacteria, require metals to maintain their metabolic, catalytic and structural functions. Vertebrate hosts have evolved several mechanisms (termed nutritional immunity) to limit the access of those pathogenic bacteria to trace metals, thus combating infections [72]. Nutritional immunity involves strategies such as metal sequestration, the redistribution of metals across cells and organ systems, and the genetic regulation of bioavailable metal levels. Collectively, these mechanisms constitute critical defenses against bacterial infection by limiting microbial access to metallic nutrients [72]. The deposition of air pollution particles containing metals in the lungs may serve as good sources of metals for microorganisms, favoring respiratory infections [24]. Furthermore, metals affect the survival and reproduction of parasites, thus influencing host–parasite interactions and the course of parasitic infections in animal populations [73,74]. Metal-induced damage to mucosal barriers also increases susceptibility to pathogens [67].

Metal exposure can cause reductions in body mass, growth and fitness [48,75]. Metabolizing toxic metals has a physiological cost [59] that can impact different aspects of animal fitness and their ability to deal with pathogens [48,76]. An exacerbated demand on the immune system, especially in the face of infectious threats, can lead to losses in other important functions, such as growth and reproduction [60].

Associations between metal levels in animals and infectious diseases may not always be directly linked to immunological factors, and confounding factors should be taken into account when interpreting results from field studies. For example, it is possible that animals living in polluted sites (and therefore more exposed to metals) may also be exposed to a greater burden and diversity of pathogens, which proliferate precisely due to the high pollution levels [29].

It is also essential to emphasize that the toxic effects of a particular metal should be evaluated within the context of its potential interactions with other elements. For example, Se, an essential trace element for the proper immune system function, exhibits a strong affinity for Hg. Consequently, Hg exposure in the context of Se deficiency can exacerbate Hg toxicity due to the reduced Se’s inhibitory effects on Hg while also impairing the activity of Se-dependent enzymes (selenoenzymes). Furthermore, Hg intoxication can result in Se retention within tissues, potentially leading to Se toxicity. On the other hand, higher Se levels in the body may reduce Hg toxicity without compromising the function of selenoenzymes. These Se–Hg interactions are particularly pronounced in brain tissues. In brief, the toxic effects of Hg and the protective role of Se in the immune system are largely influenced by the Se:Hg molar ratio [3]. This relationship underscores the complexity of metal toxicity, which is often difficult to associate with a particular health effect or outcome, especially in organisms exposed to a diverse mixture of metals, as is commonly observed in polluted environments. These aspects may help explain unexpected or difficult-to-interpret effects observed in studies involving metals, the immune system and infections in natural environments.

It is also important to consider the protective effects against metal poisoning exerted by some groups of parasites, such as acanthocephalans, on hosts. The reduced uptake of metals by hosts with a high parasite load associated with the “dilution” of the metal load among the host–parasite mass is a phenomenon called “biodilution” [42]. We speculate that this mechanism may protect the host against the toxic effects of metals on the immune system, preventing infections by other classes of pathogens, such as viruses and bacteria.

**Figure 1:**
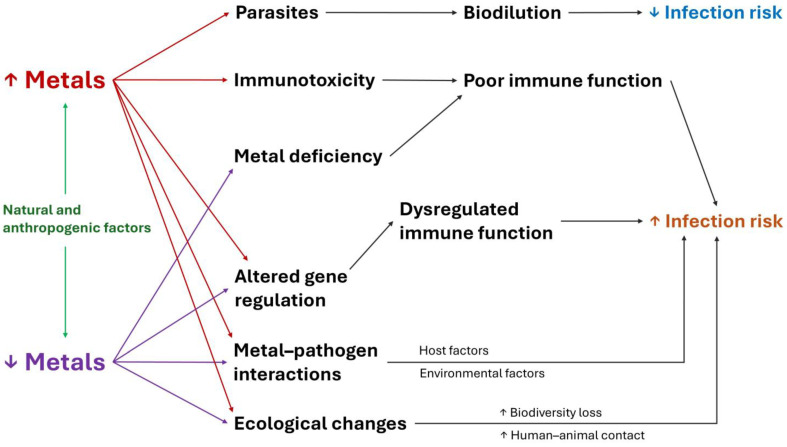
Mechanisms that explain how metals affect the risk of infectious diseases in wildlife.

Ecological mechanisms also explain the association between metals and infectious diseases. Environmental characteristics, such as temperature, humidity, pH and pollutant concentration, influence the presence and distribution of pathogens in habitats and hosts [66,77]. In this sense, metals affect the communities, structure and function of soil microbiome, which can modify disease risk [78,79,80]. Figure 1 summarizes the main immune, physiological and ecological mechanisms that explain how metals affect the risk of infectious diseases in wildlife.

**Figure 2:**
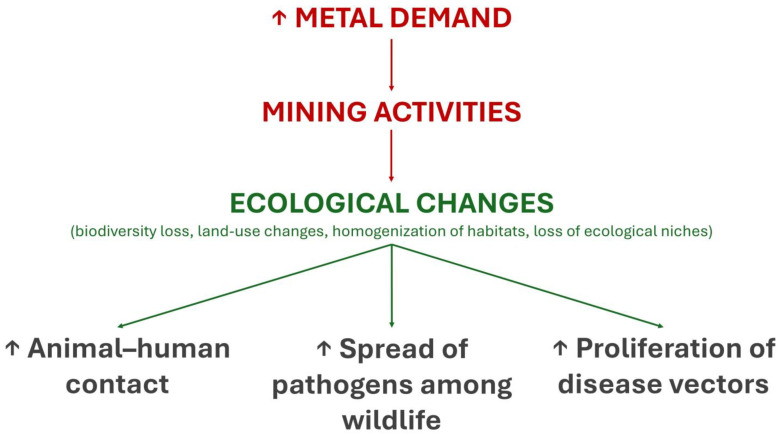
Indirect ecological effects of metals on infectious diseases.

The ecological effects of metals on infectious diseases may be indirect and must also be highlighted (Figure 2). Mining activities driven by the growing demand for metals by human societies cause ecological changes that favor the spread of pathogens. For example, deforestation in the Amazon due to mining and other anthropogenic activities promotes the loss of biodiversity, which is associated with the greater spread of pathogens among wild species [81]. Mining activities in highly biodiverse ecosystems such as the Amazon forest also increase human contact with wild animals and disease vectors (e.g., mosquitoes), which facilitates the exchange of pathogens between humans and animals, favoring both zoonoses and anthropozoonoses [82,83]. In summary, metals are at the center of direct and indirect ecological relationships that, together with their physiological and immunological effects, affect the risk of infectious diseases in different ways.

## 5. Potential Impacts of Metals on Zoonotic Spillover Events

Pollution is currently one of the main causes of ecological changes [84]. A recent meta-analysis by Mahon et al. [85] identified chemical pollution, including metal contamination, as a major factor contributing to the global emergence of infectious diseases. However, the mechanisms by which metals interfere in the emergence of infectious diseases still represent an open question. To begin unraveling this issue, it is necessary to explore the role of metals in mediating pathogen transmission, both between individual hosts and across species barriers.

Zoonotic spillover is a term commonly used to describe the transmission of a pathogenic microorganism, whether a virus, fungus, parasite or bacteria, from a wild species to the human population. Transmission can be direct (from an animal to a human) or indirect, involving the environment (animal to environment to human) or an intermediate host (animal to intermediate host, vertebrate or invertebrate, and then to human). Spillover events are the starting point for the majority (~70%) of emerging infectious diseases that affect humans. Several ecological and biological aspects of both hosts and pathogens determine the risk of spillover events occurring, such as the frequency of contact between different species, the phylogenetic distance between species and the ability of pathogens to interact with different cell types [86].

Using viral pathogens as an example, it is known that immunological factors define both the viral load of host sources and the susceptibility of recipient hosts to viral infections. For example, the episodes and amount of viral shedding of Hendra virus by bats, and consequent viral environmental contamination and spillover risk, are influenced by immunological characteristics and nutritional stress [87]. Animals under nutrient restriction-related immune deficiencies may be more susceptible to infections and favor the spread of pathogens in nature [88,89]. Humans with nutrition-related immunosuppression are more susceptible to zoonotic infections [90,91]. Individuals with Fe deficiency or overload show increased susceptibility to a variety of pathogens [92,93]. Taken together, this body of evidence shows that the susceptibility of a species to a given pathogen is influenced by nutritional/metal-related immune function, which also affects the number of pathogens expelled by hosts.

Metals may also influence the likelihood of spillover events involving parasites. In fish, greater metal availability may be associated with greater parasitemia, as discussed previously in the Section “Impacts on fish”. In red foxes, metal levels affect host interactions with *Echinococcus multilocularis*, the parasite responsible for alveolar echinococcosis, a zoonotic disease [50]. This suggests that variations in host metal levels could influence fox–parasite dynamics, potentially affecting the risk of zoonotic transmission of both known and emerging diseases to humans.

Metal-related ecological mechanisms also affect the risk of spillover events. Mineral licks are places in nature that concentrate a large quantity and variability of minerals, including metals, attracting animals that seek to supplement their diet with soil rich in these nutrients. The concentration of varied species in mineral licks facilitates the transmission of pathogens between animal species once animals deposit feces, saliva and other potentially contaminated secretions in these places, in addition to possible physical interaction between different species. Because many animals congregate there, mineral licks are also places that attract hunters, thus increasing the potential for animal–human interactions and consequently facilitating the transmission of pathogens to humans [94].

**Figure 3:**
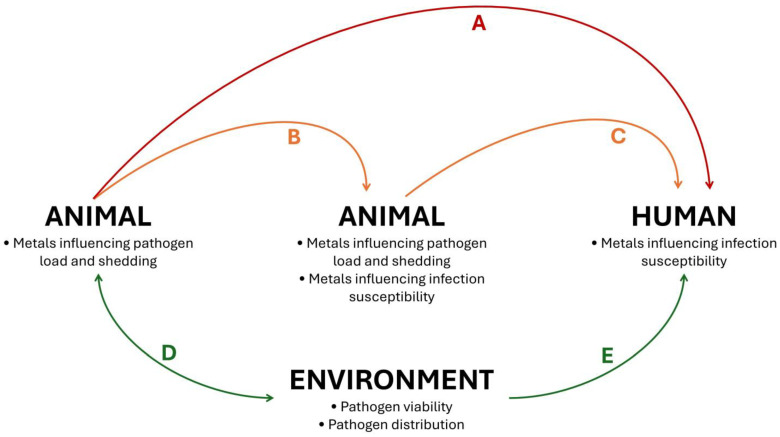
Potential effects of metals on zoonotic spillover events. Metals influence pathogen load and shedding, as well as infection susceptibility, mediating the risk of direct spillover events, without intermediate hosts (**A**) and indirect spillover events, with intermediate hosts (**B**,**C**). Furthermore, metals present in the environment affect the viability and distribution of pathogens in media such as soil and water, affecting the outcomes of environmental contamination by pathogens (**D**) and the risks of environmental-borne human infections (**E**). Of note, although the term “zoonotic spillover” is used in different contexts in the literature, in this figure and review the expression refers to the transmission of pathogens from wildlife to humans.

Considering (I) the critical and multiple immunoregulatory roles of metals on immune function in both animals and humans, (II) the variable amount of both essential and toxic metals found in the environment and (III) the direct and indirect ecological influences of metals on pathogen–animal–human interactions, we suggest that metals may affect the risk of pathogen spillover and disease emergence. In this sense, Figure 3 summarizes how metals can affect the main models of zoonotic spillover events. Studies evaluating the effects of specific metals, as well as metal mixtures, on zoonotic diseases are essential to clarify the relationship between metal exposure and zoonotic spillover.

## 6. Discussion

The effects of toxic metals are commonly studied from a human health-centered perspective, often overlooking the multiple negative impacts of metal pollution on wildlife. The Minamata Bay disaster is a notable example of Hg pollution’s impact on wildlife. The industrial discharge of Hg compounds into the Japanese bay from 1932 to 1968 led to the widespread formation of toxic methylmercury, which bioaccumulated in fish and shellfish as a result of environmental pollution [95,96]. This contamination caused severe neurological damage and mortality in marine animals, terrestrial species such as cats and predatory birds, resulting in population declines [95,96]. Of note, Minamata disease, the term for the human health issues caused by Hg poisoning, was first identified only in 1956, after children began exhibiting Hg-related neurological symptoms [96]. This highlights the high degree of permanence and bioaccumulation of Hg in the environment, in addition to drawing attention to the fact that metal pollution has been affecting humans and animals for many decades. The Minamata Bay disaster emphasizes the importance of considering wildlife effects in heavy metal pollution assessments. Our review expanded the understanding of the effects of metals on wildlife by providing a discussion of how metals influence the dynamics of infectious diseases.

Studying the effects of metals on infectious processes is complex, especially in natural environments. Pollutants can have varying effects on infectious diseases, increasing or decreasing the risk of infection in different biological contexts [52]. For example, in bats, Hg can impair proper immune function and facilitate infection. On the other hand, Hg can, through its cellular toxic effects, decrease the number of cells available for pathogens to infect, thus decreasing the infection rate. Reduced infection rates can also be explained by the direct toxic effects of Hg on the survival of pathogens. Furthermore, these effects may vary between different bat taxa [52]. Indeed, the studies reviewed in this paper showed varying effects of metals on different species (Table 2).

Our review also explored the potential mechanisms by which metals may affect the risk of spillover events (Figure 3), highlighting that the influence of metals on infection dynamics in wildlife species may reverberate over human health. In addition to the direct impacts of zoonoses on human populations (e.g., deaths, morbidity, epidemics), the burden of infectious diseases triggered by metal-induced spillover events may add to the burden of chronic diseases caused by metal pollution around the world. For example, the neurotoxic effects of Pb may have compounded the neuroimmune effects of SARS-CoV-2 during the COVID-19 pandemic, significantly impairing children’s development in socially vulnerable communities [97].

The complexity of studying metal–infection relationships increases even further when more than one metal is evaluated together. Although challenging, this multi-metal approach is important because, in nature, especially in highly anthropized environments, organisms are often exposed to mixtures of metals [71]. Another aspect that needs to be taken into consideration is related to the dose of each metal to which the organisms were exposed and the particularities of the tolerance of each organism. Using humans as an example, the safe dose range (i.e., exposure through food) for essential elements such as Fe and Zn is relatively wide [98,99]; thus, it is not common to observe deleterious effects caused by exposure to these metals. However, for other non-essential elements, such as Pb, no exposure dose is considered safe due to the high toxic effects of this metal [100]. These complexities related to exposure dose are particular to each species, and little is known about metal tolerance and poisoning effects (e.g., safety doses, sub-lethal outcomes) in non-human animals. Furthermore, different chemical forms of the same metal can exhibit distinct toxicological effects on cells and biological systems, including the immune system. These variations further complicate the interpretation and comparison of findings across studies that investigate different compounds of the same metal [71]. These limitations and biological factors must be taken into account when interpreting studies involving metals and infectious diseases.

Our review showed that metal pollution impacts different places around the world, especially regions with mining activities (Table 1). Therefore, the remediation of metal-contaminated soils is essential to mitigate the negative impacts on ecosystems. Conventional remediation methods for metal-contaminated soils (e.g., vitrification, soil incineration, excavation and landfill, soil washing, soil flushing, solidification and stabilization, electro-kinetic systems) often involve high costs, energy consumption, and potential secondary pollution [101,102]. In contrast, phytoremediation has emerged as a cheaper and environmentally friendly alternative, offering a “green” solution to mitigate metal pollution through the use of plants to extract, stabilize or degrade contaminants in the soil and water [101].

Different types of phytoremediation methods can be applied to reduce metal contamination in soil and water, including phytoextraction, phytovolatilization, phytostabilization and phytofiltration. These methods are based on distinct physiological processes performed by plants [103]. Depending on the type and form of contaminants, as well as the medium involved, different plant species may utilize one or a combination of these mechanisms to effectively remediate polluted environments [104]. The plant species *Noccaea caerulescens*, *Noea mucronata*, *Prosopis laevigata*, *Siegesbeckia orientalis*, *Sedum plumbizincicola*, *Alyssoides utriculata* and *Plantago major* are considered metal hyperaccumulators, and are potentially good choices for phytoremediation strategies [105]. Moreover, plant physiology and the success of phytoremediation are influenced not only by abiotic factors such as soil properties and climate but also by biotic interactions with insects, pests, and microorganisms. These factors need to be considered in phytoremediation strategies. Additionally, site management practices play a crucial role in determining the overall outcome [106].

Phytoremediation depends on the establishment and growth of vegetation, which in turn requires favorable conditions such as a suitable climate, soil type and agronomic practices, and the careful selection of plant species. Considering that soil characteristics and weather conditions vary significantly between sites, the effectiveness of phytoremediation observed with a particular plant species in one location may not be replicated elsewhere, making the process highly site-specific [106]. In this sense, it is recommended to use plant species native or “naturalized” to the region being remediated [105]. Additionally, phytoremediation generally requires a longer time and tends to be less effective compared to conventional remediation methods. However, its efficiency can be improved through genetic engineering, and the use of beneficial microorganisms and soil amendments [106]. Therefore, a comprehensive understanding of these multiple factors is essential to optimize phytoremediation strategies and enhance their effectiveness in diverse environmental conditions.

In addition to strategies focused on remediating metal pollution, it is necessary to reduce the amount of metals released into the environment. This is the most relevant action to reduce metal pollution in ecosystems. This goal can be achieved by developing mining technologies that generate less mining waste. However, the most needed action is to reduce society’s demand for metals/mining by promoting more sustainable lifestyles, which involve reducing consumption patterns, improving public transportation and recycling electronic products that contain metals. In other words, it is necessary to promote economic systems that value social well-being and healthy human development rather than focusing on economic development as the main objective [107,108]. Moreover, considering the metal load present in pesticides [5,109], it is required to strengthen projects focused on the production of foods and other agricultural products that are free of pesticides, such as agroecological systems and organic “family” farming, limiting the expansion of monocultures and other forms of production that degrade ecosystems and contribute to metal pollution globally. Finally, it is important to invest in monitoring metals in humans, the environment and animals in order to identify the locations and species most vulnerable to metal pollution through a One Health approach. These strategies will help reduce the undesirable effects of metals on infectious diseases in an integrated and realistic way.

## 7. Conclusions

As evidenced in Table 2 and discussed throughout this article, there is a clear need for further studies investigating how metals influence the risk of infections in animals living in natural environments, especially for certain animal groups, such as insects and reptiles. These studies are essential to add pieces to the puzzle of zoonotic diseases in the Anthropocene, when metal pollution is increasing rapidly, harming wildlife and humans.

Exposure to toxic metals from the environment or through food sources impairs the immune system of different animal species, facilitating infectious processes in natural environments. Poor immune function caused by nutritional deficiencies in essential metals also facilitates infections. Other metal-mediated mechanisms, such as the regulation of immune system genes, host–pathogen interactions and metal-induced damage to mucosal barriers, could also explain how metals influence the risk of infectious processes. Interestingly, some parasites can protect hosts from metal toxicity through the process of biodilution.

In addition to physiological and immunological mechanisms, direct and indirect ecological processes explain, at least in part, the association between metals and infectious diseases. We suggest that animal exposure to toxic metals may facilitate zoonotic spillover events and the emergence of new diseases in human populations. Moreover, due to the complex interactions between metals, the environment and different animals, as well as considering that both low and high levels of a given element can affect the host immune system, the identification of ranges of exposure and the association between such ranges and specific outcomes is mandatory to obtain a better comprehension of metal-related infectious processes. Finally, controlling metal pollution is essential to preserve the health of humans and non-human animals, in addition to protecting ecosystems.

## Figures and Tables

**Table 1:** Examples of pollution caused by mining activities in various regions of the world.

Region and/or Country	Mining Enterprise	Type of Pollution	Reference
Hunan, China	Manganese mining	Metal pollution	Luo et al. [12]
Zambia and the Democratic Republic of Congo	Copper–cobalt mining	High concentrations of trace elements	Muimba-Kankolongo et al. [13]
Odisha, India	Chromite mining	Chromium pollution	Das et al. [14]
Huelva, Spain	Copper mining	Acid mine drainage	Olías and Nieto [15]
New Brunswick, Canada	Copper–zinc mining	Copper–zinc pollution	Saunders and Sprague [16]
Myanmar and other Southeast Asian countries, Asia	Artisanal and small-scale gold mining	Mercury pollution	Soe et al. [17]
Tasmania, Australia	Copper mining	Metal pollution	Beck et al. [18]
Witwatersrand basin, South Africa	Gold mining	Uranium and other metals pollution	Winde and Sandham [19]
Kratovo-Zletovo and Sasa-Toranica, Republic of North Macedonia	Lead–zinc mining	High concentrations of trace elements	Ramani et al. [20]
Attica, Greece	Silver mining	Metal pollution	Stamatis et al. [21]
	Lead–zinc mining and smelting industrial complex	Lead pollution	Maravelias et al. [22]

**Table 2:** Main effects of metals on infections in animals sampled in nature.

Animal Group	Popular Name	Main Findings	Reference
Insect	Bumblebee	Although bees from a specific polluted site exhibited a higher infection rate, metal pollution indexes showed no significant correlation with *Nosema bombi* infection levels.	Szentgyörgyi et al. [34]
Mollusk	Periwinkle	*Littorina littorea* periwinkles infected with digenean parasites showed reduced metal levels (i.e., Fe, Cu, Ni) compared to uninfected periwinkles.	Evans et al. [35]
	Cockles	No major significant difference in metal levels between cockles parasitized with *Labratrema minimus* and those without infection.	Baudrimont et al. [36]
	Snail	Cadmium, Pb, Cu and Hg water levels affected the *Schistosoma mansoni* infection rate in the offspring of *Biomphalaria alexandrina* snails (the snails were collected in water bodies in nature, but snails were reared in the laboratory and their first generations were used in infection experiments).	Sharaf El-Din et al. [37]
Fish	Atlantic salmon	A bacterial epizootic outbreak (*Aeromonas liquefaciens*) affecting Atlantic salmon (*Salmo salar*) and suckers (*Catostomus commersonii*) was associated with metal pollution (Cu and Zn) and high river temperatures.	Pippi and Hare [38]
	Marbled spinefoot	Cadmium and Pb concentrations in the liver of fish (*Siganus rivulatus*) infected with *Sclerocollum rubrimaris* were lower compared to uninfected fish.	Hassanine and Al-Hasawi [39]
	Chub	Chub (*Squalius cephalus*) infected with *Pomphorhynchus laevis* showed lower levels of Cu, Cd and Pb compared to uninfected fish.	Marijić et al. [40]
	Common carp and sharptooth catfish	Common carp (*Cyprinus carpio*) infected with the cestode *Atractolytocestus huronensis* showed a tendency of lower metal concentrations compared to uninfected fish. Sharptooth catfish (*Clarias gariepinus*) with a higher infection load by the nematode *Contracaecum* sp. showed lower tissue levels of metals compared with those fish with reduced parasite load.	Erasmus et al. [41]
	Brown trout	Acanthocephalans (*Dentitruncus truttae*) showed higher metal content (e.g., As, Cr, Mn, Pb, Cd, Tl) than fish (*Salmo trutta*) in intestinal tissues. A higher parasite load was associated with lower metal accumulation in fish.	Mijošek et al. [42]
Reptile	Lizard	Nematode and cestode infection intensity in *Chalcides ocellatus* lizards correlated positively with Cu, Cd and Pb levels.	Soliman [43]
Bird	Sparrow	Lead concentrations were positively correlated with *Plasmodium relictum* prevalence in *Passer domesticus* sparrows. Cadmium was negatively associated with infection prevalence.	Bichet et al. [29]
		Elevated Pb levels were associated with increased gapeworm infection rate. Mercury also showed moderate association. A lower concentration of V was observed in infected birds.	Khan et al. [44]
	Oldsquaw	High levels of Cd in tissues were associated with an outbreak of avian cholera in oldsquaws (*Clangula hyemalis*). Selenium and Hg liver concentrations were lower in infected birds.	Mashima et al. [45]
	Pigeon	High levels of Zn in feathers were associated with protection against *Chlamydiaceae* and Haemosporidian pathogens in urban pigeons (*Columba livia*). Elevated Pb levels were associated with increased Haemosporidian infection intensity.	Gasparini et al. [46]
Mammal	Harbor porpoise	Harbor porpoises (*Phocoena phocoena*) that died from infectious diseases had higher average liver metal levels compared to controls that died from physical trauma.	Bennet et al. [47]
	Wood mice	Resistance of wood mice (*Apodemus sylvaticus*) to parasites decreased with increasing exposure to metals.	Tersago et al. [48]
	Rodents	The classes of parasites with which small rodents (*Microtus agrestris* and *Apodemus flavicolis*) are infected (cestodes: *Paranoplocephala* spp. versus nematodes: *Mastophorus muris*) influence the levels of metals in rodent tissues.	Jankovská et al. [49]
	Red fox	Foxes (*Vulpes vulpes*) infected with *Echinococcus multilocularis* showed reduced levels of Cd and Pb and increased levels of other metals, especially Fe, compared to non-infected foxes.	Brožová et al. [50]
		Foxes (*Vulpes vulpes*) infected with the intestinal parasites (*Toxascaris leonina* and *Mesocestoides* spp.) showed decreased Pb levels compared to non-infected foxes. The levels of other metals were also affected by infection status.	Jankovská et al. [51]
	Bat	Mercury was associated with varying bacterial infection outcomes across bat species within a bat community in Belize.	Becker et al. [52]

## Data Availability

No new data were created or analyzed in this study.
